# Toward literature-based feature selection for diagnostic classification: a meta-analysis of resting-state fMRI in depression

**DOI:** 10.3389/fnhum.2014.00692

**Published:** 2014-09-10

**Authors:** Benedikt Sundermann, Mona Olde lütke Beverborg, Bettina Pfleiderer

**Affiliations:** Department of Clinical Radiology, University Hospital MünsterMünster, Germany

**Keywords:** depression, depressive disorder, functional neuroimaging, magnetic resonance imaging, meta-analysis, feature selection

## Abstract

Information derived from functional magnetic resonance imaging (fMRI) during wakeful rest has been introduced as a candidate diagnostic biomarker in unipolar major depressive disorder (MDD). Multiple reports of resting state fMRI in MDD describe group effects. Such prior knowledge can be adopted to pre-select potentially discriminating features for diagnostic classification models with the aim to improve diagnostic accuracy. Purpose of this analysis was to consolidate spatial information about alterations of spontaneous brain activity in MDD, primarily to serve as feature selection for multivariate pattern analysis techniques (MVPA). Thirty two studies were included in final analyses. Coordinates extracted from the original reports were assigned to two categories based on directionality of findings. Meta-analyses were calculated using the non-additive activation likelihood estimation approach with coordinates organized by subject group to account for non-independent samples. Converging evidence revealed a distributed pattern of brain regions with increased or decreased spontaneous activity in MDD. The most distinct finding was hyperactivity/hyperconnectivity presumably reflecting the interaction of cortical midline structures (posterior default mode network components including the precuneus and neighboring posterior cingulate cortices associated with self-referential processing and the subgenual anterior cingulate and neighboring medial frontal cortices) with lateral prefrontal areas related to externally-directed cognition. Other areas of hyperactivity/hyperconnectivity include the left lateral parietal cortex, right hippocampus and right cerebellum whereas hypoactivity/hypoconnectivity was observed mainly in the left temporal cortex, the insula, precuneus, superior frontal gyrus, lentiform nucleus and thalamus. Results are made available in two different data formats to be used as spatial hypotheses in future studies, particularly for diagnostic classification by MVPA.

## Introduction

Mental disorders featuring depression as a predominant symptom and more specifically major depressive disorder (MDD) are important worldwide public health concerns. In recent years significant progress has been achieved regarding the identification of biological correlates and potential neural mechanisms involved in the pathogenesis of MDD. These scientific efforts comprise studies of genetic foundations, molecular mechanisms including neurotransmitter systems and structural as well as functional neuroimaging (Kupfer et al., 2012). Thereby candidate neural systems have been identified that support emotion processing, reward seeking, regulate emotion and are therefore presumed to play an important role in MDD. These networks include subcortical as well as cortical (particularly prefrontal and cingulate) brain regions modulated by serotonin and dopamine neurotransmission (Kupfer et al., 2012).

A majority of reported functional magnetic resonance imaging (fMRI) studies in MDD has applied stimulus-based acquisition protocols. Participants were confronted with predefined stimuli in the scanner, e.g., pictures of emotional faces. Brain activity in response to these stimuli was analyzed (Fitzgerald et al., 2008; Stuhrmann et al., 2011; Delvecchio et al., 2012; Diener et al., 2012; Groenewold et al., 2013). Stimulus-based fMRI requires rather complex experimental setups. In contrast, fMRI at rest, so-called resting-state fMRI (rs-fMRI), facilitates the examination of spontaneous neural activity in networks that highly resemble those observed in task-based fMRI (Smith et al., 2009). It necessitates simpler, but nonetheless highly standardized data acquisition procedures (Van Dijk et al., 2010) and has therefore attracted attention by researchers interested in clinical applications of fMRI (Zhang and Raichle, 2010; Lee et al., 2012; Barkhof et al., 2014; Sundermann et al., 2014a).

A broad methodological spectrum for analyses of rs-fMRI data has been developed and there is no standard analysis strategy for group comparisons either. This heterogeneity is reflected in rs-fMRI studies in MDD as well. However, most analyses are either based on regional features or on connectivity of distant brain regions. Typical regional measures include regional homogeneity (ReHo) or the (fractional) amplitude of characteristic low-frequency fluctuations (ALFF or fALFF). Functional connectivity (FC) can be operationalized as the temporal correlation of signal fluctuations in remote brain areas. Conventional FC-analyses are seed-based but FC-analyses in a wider sense include independent component analyses or complex graph theoretical network measures. A minority of studies has applied analyses of effective connectivity (such as Granger causality taking temporal dependencies into account) (Margulies et al., 2010; van den Heuvel and Hulshoff Pol, 2010). Rs-fMRI is increasingly adopted scientifically in subjects with MDD (Wang et al., 2012b; Kühn and Gallinat, 2013). Despite the qualitative similarity of networks observed during task-fMRI and rs-fMRI it has not been firmly established which features of stimulus-related neural correlates of MDD can be sufficiently captured by rs-fMRI. The exact relation of rs-fMRI and other neuroimaging methods at rest including positron emission tomography (PET) is still subject to ongoing research as well (Chetelat et al., 2013; Riedl et al., 2014).

Whereas most neuroimaging studies in MDD focus on disease mechanisms at the group level, there is substantial interest in identifying biomarkers that are clinically applicable as diagnostic tools in single subjects (Mossner et al., 2007; Atluri et al., 2013; Schneider and Prvulovic, 2013). Particularly, important recent approaches for diagnostic classification in various mental disorders are based on the combination of rs-fMRI with multivariate pattern analysis techniques (MVPA) (Klöppel et al., 2011; Orru et al., 2012; Zarogianni et al., 2013; Haller et al., 2014; Sundermann et al., 2014a). MVPA subserves the automated generation of decision rules based on previous experience, labeled training data in this particular case. MVPA approaches integrate information from multiple brain regions with the aim to increase diagnostic power compared to conventional univariate analysis schemes that are used in many fMRI studies (Pereira et al., 2009; Sundermann et al., 2014a). Seminal work in the field of exploring the clinical applicability of rs-fMRI in combination with MVPA has been done in subjects with MDD (Craddock et al., 2009).

Functional neuroimaging data are typically rather noisy and high-dimensional. Therefore, different feature selection (FS) methods have been proposed to identify a subset of most informative features to be used with the aim to increase classification accuracy (Pereira et al., 2009). There is a fundamental distinction between FS approaches using prior knowledge (Chu et al., 2012) and data-driven methods, particularly filters or wrappers, that use the training dataset itself for FS (Pereira et al., 2009; Mwangi et al., 2013). Recent evidence from structural neuroimaging in dementia indicates that FS based on prior knowledge may be advantageous. In that report support vector machines (SVM) were used for classification (Chu et al., 2012). Such kernel methods like SVM are especially popular in recent attempts to classify fMRI datasets (Orru et al., 2012; Sundermann et al., 2014a).

There seems to be a substantive body of scientific studies on rs-fMRI in MDD now. However, methods of data analysis and results are very heterogeneous. Previous efforts to specifically summarize these rs-fMRI findings have focused on specificity and interpretability regarding disease mechanisms and therefore conducted rather exclusive study selection (only five rs-fMRI studies finally included) and pooled studies with SPECT and PET data (Kühn and Gallinat, 2013) or adopted qualitative methods of data synthesis (Wang et al., 2012b). Consequently, they are not optimally suited to select brain areas that contain particularly important information for FS used later to enable clinical differentiation in MDD by MVPA.

Purpose of this meta-analysis is to consolidate spatial information about alterations of spontaneous brain activity in patients with unipolar depression compared to healthy controls. This investigation is specifically intended to generate and make available “prior knowledge” that can be readily used as spatial hypotheses in rs-fMRI studies in MDD, particularly studies applying machine-learning methods for diagnostic classification. This includes but is not limited to pre-selection of features for diagnostic MVPA approaches. For this reason spatial precision and sensitivity are priorized over functional interpretability regarding exact disease mechanisms.

## Materials and methods

### Identification and selection of relevant studies

We conducted a PubMed (http://www.ncbi.nlm.nih.gov/pubmed/) search using the following query on August 20, 2013: (*“depression” OR “depressive”) AND (“fMRI” OR “functional MRI” OR “functional magnetic”) AND (“functional connectivity” OR “resting state” OR “resting-state”*).

Initially, 183 results were identified. In addition, we also screened a recent review for further papers (Wang et al., 2012b) and a prior rather exclusive meta-analysis (Kühn and Gallinat, 2013) comprising rs-fMRI alterations in depression. Thereby six additional articles were identified. Titles and abstracts were manually screened twice (by two individuals) for studies (in English language) reporting results on rs-fMRI in adult patients with typical subtypes of unipolar depression (not adolescent, postpartum and late-life depression as well as studies that aimed at investigating a specific comorbidity) compared to healthy controls. Of these 51 studies identified, whole text versions were screened for studies fulfilling these criteria as well as including at least 10 subjects per group and reporting resulting coordinates of group comparisons (depression vs. healthy controls) in either MNI/ICBM (Mazziotta et al., 2001) or Talairach (1988) space. Thirty Two studies fulfilled these criteria and were therefore included in the final analyses. Studies by the same authors were screened for highly similar demographical characterization of samples and were otherwise considered independent in further analyses based on consensus of all three authors.

### Coordinate-based meta-analysis

Reported maxima coordinates were extracted and, if reported in Talairach space, converted to MNI space using tal2icbm (Lancaster et al., 2007; Laird et al., 2010). As an exception, coordinates from one study (Lui et al., 2011) were transformed using tal2mni (Brett et al., 2001) as final coordinates in that study had initially been transformed using this method. Coordinates were assigned to two categories based on directionality of findings in order to avoid that clearly opposed findings in the original studies enhance each other in the ALE-analysis: group A comprises findings of decreased long distance or local connectivity (including lower correlation coefficients or lower regional homogeneity) or lower power of typical low frequency fluctuations representing spontaneous neural activity in depression compared to healthy controls and findings without clearly interpretable directionality information (Greicius et al., 2007; Bluhm et al., 2009; Yao et al., 2009; Liu et al., 2010, 2013a,b; Veer et al., 2010; Zhou et al., 2010; Furman et al., 2011; Guo et al., 2011a,b, 2012a,b, 2013a,b,c; Hamilton et al., 2011; Lui et al., 2011; Peng et al., 2011, 2012; Wu et al., 2011; Ma et al., 2012, 2013; Wang et al., 2012a, 2013a,b; Ye et al., 2012; Zhu et al., 2012; Tang et al., 2013; Zeng et al., 2013). Details of these studies are presented in Table 1. Group B represents increased connectivity or low frequency fluctuations in depression compared to controls (Table 2) (Liu et al., 2010, 2013a,b; Sheline et al., 2010; Veer et al., 2010; Zhou et al., 2010; Furman et al., 2011; Guo et al., 2011a,b, 2012b, 2013a,b; Hamilton et al., 2011; Wu et al., 2011; Cao et al., 2012; Ma et al., 2012; Wang et al., 2012a, 2013a; Ye et al., 2012; Zhu et al., 2012).

**Table 1:** **Studies in group A (representing mainly decreased connectivity/function in depression and ambiguous directionality)**.

**Sample number**	**Author and year**	**Samle size and depression subtype**	**Medication**	**Primary analysis method**
1	Wang et al., 2013a	14 (MDD, first episode), 14 (HC)	Partially	ReHo
	Wang et al., 2013b	17 (MDD, first episode), 17 (HC)	No	VMHC
	Wang et al., 2012a	18 (MDD, first episode), 18 (HC)	No	(f)ALFF
2	Guo et al., 2013c	22 (MDD, treatment resistant), 23 (MDD, treatment sensitive), 19 (HC)	Yes	VMHC
	Guo et al., 2012a	22 (MDD, treatment resistant), 23 (MDD, treatment sensitive), 19 (HC)	Yes	ReHo-based
	Liu et al., 2013b	22 (MDD, first episode), 19 (HC)	No	fALFF
3	Guo et al., 2013b	24 (MDD, first episode), 24 (HC)	No	fALFF, Seed-FC (Cerebellum)
	Guo et al., 2013a	24 (MDD; first episode), 24 (HC)	No	VMHC
4	Zeng et al., 2013	24 (MDD), 29 (HC)	No	Seed-FC (anterior cingulate)
	Ma et al., 2013	24 (MDD), 29 (HC)	No	Seed-FC (cerebellum)
5	Liu et al., 2013a	22 (MDD), 26 (HC)	Yes	fALFF
6	Tang et al., 2013	28 (MDD), 30 (HC)	No	Seed-FC (amygdala)
7	Peng et al., 2012	16 (MDD), 16 (HC)	No	Seed-FC (anterior cingulate)
	Peng et al., 2011	16 (MDD), 16 (HC)	No	ReHo
8	Ma et al., 2012	18 (MDD, treatment resistant), 17 (MDD, treatment sensitive) 17 (HC)	Yes	Seed-FC (based on gray matter abnormalities)
	Guo et al., 2012b	18 (MDD, treatment resistant), 17 (MDD, treatment sensitive) 17 (HC)	Yes	ALFF
9	Ye et al., 2012	22 (MDD, first episode), 30 (HC)	No	Seed-FC (right DLPFC)
10	Zhu et al., 2012	35 (MDD, first episode), 35 (HC)	No	ICA
11	Guo et al., 2011a	17 (MDD), 17 (HC)	Yes	ReHo
	Guo et al., 2011b	24 (MDD, treatment resistant) 19 (MDD, treatment resistant)	Yes	ReHo
12	Furman et al., 2011	21 (MDD, women only), 19 (HC, women only)	Yes	Seed-FC (striatum)
13	Veer et al., 2010	19 (MDD), 19 (HC)	No	ICA
14	Wu et al., 2011	22 (MDD, treatment resistant), 26 (HC)	Yes	ReHo
15	Liu et al., 2010	14 (MDD), 15 (HC)	No	ReHo
16	Hamilton et al., 2011	16 (MDD), 14 (HC)	No	Granger causality
17	Bluhm et al., 2009	14 (MDD), 15 (HC)	No	Seed-FC (precuneus/posterior cingulate cortex)
18	Yao et al., 2009	22 (MDD), 22 (HC)	Partially	ReHo
19	Greicius et al., 2007	28 (MDD), 20 (HC)	Yes	ICA
20	Lui et al., 2011	32 (MDD, treatment sensitive), 28 (MDD, treatment resistant), 48 (HC)	Yes	Seed-FC (multiple)
21	Zhou et al., 2010	18 (MDD), 20 (HC)	No	Seed-FC (multiple)

*Individual reports are grouped by samples according to potential overlap*.

*HC, healthy controls; MDD, major depressive disorder; FC, functional connectivity; ReHo, regional homogeneity; (f)ALFF, (fractional) amplitude of low frequency fluctuations; VMHC, voxel-mirrored homotopic connectivity; ICA, independent component analysis; DLPFC, dorsolateral prefrontal cortex*.

**Table 2:** **Studies in group B (representing mainly increased connectivity/function in depression)**.

**Sample number**	**Author and year**	**Sample size and depression subtype**	**Medication**	**Primary analysis method**
1	Wang et al., 2013a	14 (MDD, first episode), 14 (HC)	Partially	ReHo
	Wang et al., 2012a	18 (MDD, first episode), 18 (HC)	No	(f)ALFF
2	Guo et al., 2013b	24 (MDD, first episode), 24 (HC)	No	fALFF, Seed-FC (Cerebellum)
	Guo et al., 2013a	24 (MDD, first episode), 24 (HC)	No	VMHC
3	Liu et al., 2013a	22 (MDD), 26 (HC)	Yes	fALFF
4	Liu et al., 2013b	22 (MDD, first episode), 19 (HC)	No	fALFF
5	Peng et al., 2012	16 (MDD), 16 (HC)	No	Seed-FC (anterior cingulate)
6	Ma et al., 2012	18 (MDD, treatment resistant), 17 (MDD, treatment sensitive) 17 (HC)	Yes	Seed-FC (based on gray matter abnormalities)
	Guo et al., 2012b	18 (MDD, treatment resistant), 17 (MDD, treatment sensitive) 17 (HC)	Yes	ALFF
7	Ye et al., 2012	22 (MDD, first episode), 30 (HC)	No	Seed-FC (right DLPFC)
8	Cao et al., 2012	42 (MDD), 32 (HC)	No	Seed-FC (hippocampus)
9	Zhu et al., 2012	35 (MDD, first episode), 35 (HC)	No	ICA
10	Guo et al., 2011a	17 (MDD), 17 (HC)	Yes	ReHo
	Guo et al., 2011b	24 (MDD, treatment resistant) 19 (MDD, treatment resistant)	Yes	ReHo
11	Furman et al., 2011	21 (MDD, women only), 19 (HC, women only)	Yes	Seed-FC (striatum)
12	Veer et al., 2010	19 (MDD), 19 (HC)	No	ICA
13	Wu et al., 2011	22 (MDD, treatment resistant), 26 (HC)	Yes	ReHo
14	Sheline et al., 2010	18 (MDD), 17 (HC)	No	Seed-FC (multiple)
15	Liu et al., 2010	14 (MDD), 15 (HC)	No	ReHo
16	Hamilton et al., 2011	16 (MDD), 14 (HC)	No	Granger causality
17	Zhou et al., 2010	18 (MDD), 20 (HC)	No	Seed-FC (multiple)

*Individual reports are grouped by samples according to potential overlap*.

*HC, healthy controls; MDD, major depressive disorder; FC, functional connectivity; ReHo, regional homogeneity; (f)ALFF, (fractional) amplitude of low frequency fluctuations; VMHC, voxel-mirrored homotopic connectivity; ICA, independent component analysis; DLPFC, dorsolateral prefrontal cortex*.

Coordinate-based meta-analyses were calculated with GingerALE (Research Imaging Institute, University of Texas Health Science Center, San Antonio, TX, USA, version 2.3.1, http://www.brainmap.org/ale/) using the non-additive activation likelihood estimation (ALE) approach with coordinates organized by subject group (ALE-S method) to account for non-independent samples (Turkeltaub et al., 2012). ALE-S is an extension of the random effects ALE approach (Eickhoff et al., 2009) that prevents multiple experiments performed by one subject group from cumulatively influencing ALE values. Therefore, a modeled activation map is generated for each subject group independently based on published coordinates in a first step. These maps are then combined in a second step to calculate final ALE values (Turkeltaub et al., 2012). Coordinates in group A were assigned to 21, in group B to 17 presumably independent subjects groups as indicated in Tables 1, 2. Coordinates were masked using the conservative standard mask in Ginger ALE. 11 locations in group A and 7 locations in group B were located outside the mask while 305 (group A) and 132 (group B) foci remained inside. Study specific smoothing using a Gaussian kernel (group A: FWHM median = 9.17 mm, range 8.88–9.57 mm, group B: FWHM median = 9.28 mm, range 8.87–9.50 mm) was applied based on the mean sample size per subject group to take different sample sizes into account. Results were thresholded at *p* < 0.05 corrected for multiple comparisons using cluster-based correction with a cluster-forming threshold of *p* < 0.01 (uncorrected) and 1000 permutations (Eickhoff et al., 2012) resulting in a minimum cluster size of 528 mm^3^ in group A and 544 mm^3^ in group B. All analyses were calculated in MNI space.

Anatomical labels were automatically assigned in GingerALE. Visualizations were created using Mango (Research Imaging Institute, University of Texas Health Science Center, San Antonio, TX, USA, version 3.0.4, http://ric.uthscsa.edu/mango/) and a high resolution anatomical template with isotropic voxels in MNI space as distributed with GingerALE.

## Results

### Decreased or ambiguously altered spontaneous functional connectivity/activity in MDD

**Figure 1:**
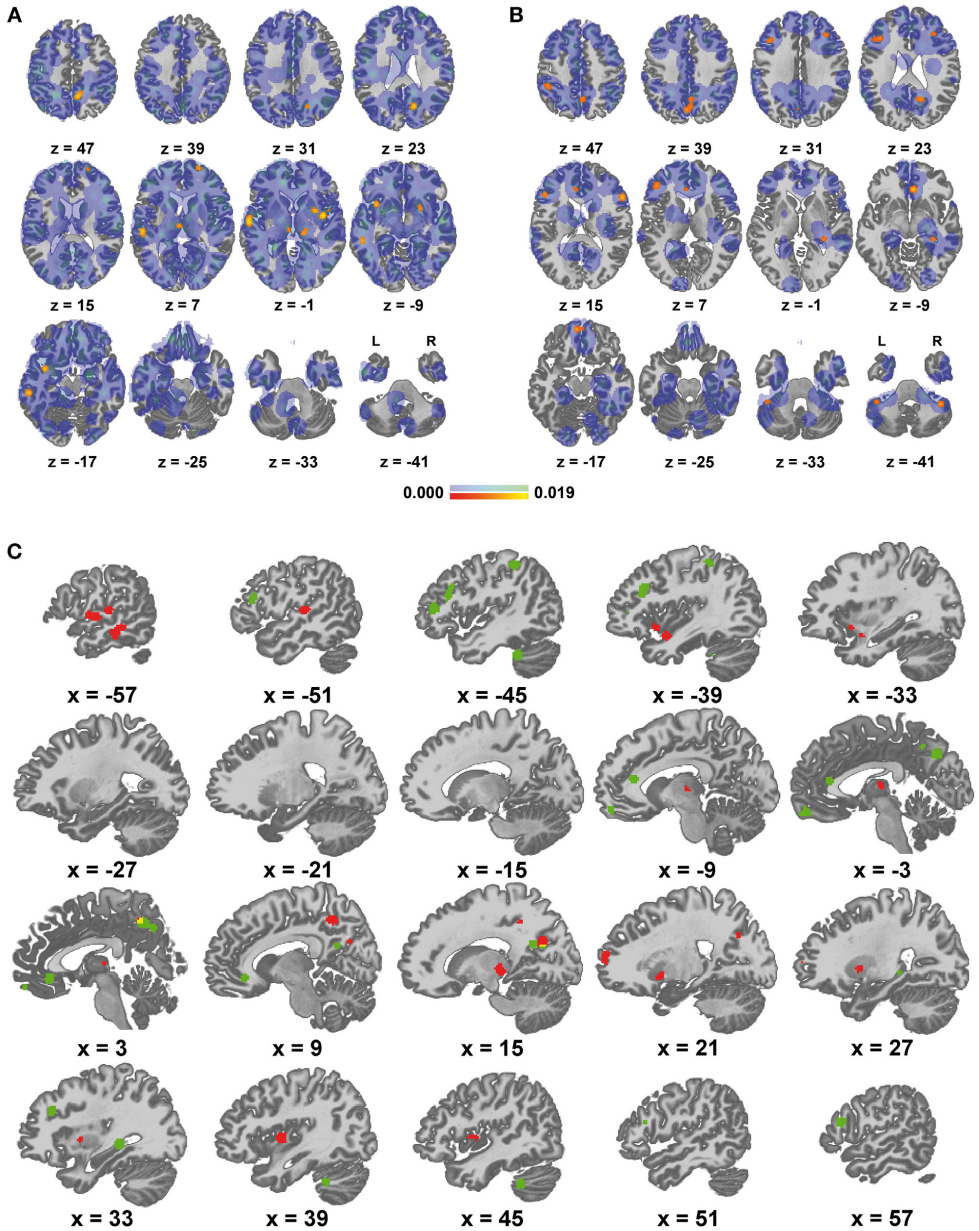
**Areas of altered functional connectivity/activity in depression compared to controls**. **(A)** Red to yellow: significant meta-analytic results (*p* < 0.05) in group A (representing mainly decreased connectivity / function in depression), blue to green: unthresholded ALE values, **(B)** equivalent representation of group B (increased connectivity/function in depression), **(C)** qualitative display of significant results, red: group A, green: group B, yellow: overlap.

Results of group A spatially converged mainly in the left superior/middle temporal gyrus and bilaterally in the insula, precuneus, superior frontal gyrus, lentiform nucleus and thalamus. For detailed results see Figures 1A,C and Table 3. Complete thresholded ALE-maps are made available in NIfTI-1 data format as Supplementary Material.

**Table 3:** **Brain areas (cluster-information and peak voxels) with significant convergence across studies in Group A (mainly decreased connectivity/activity in depression)**.

**Anatomical label**	**BA**	**(Sub-)Maxima coordinates**			**ALE**
		***x***	***y***	***z***	
**Cluster 1 (4 contributing subject groups, volume: 1048 mm^3^, weighted center: *x* = −59, *y* = −9, *z* = 2)**					
Left superior temporal gyrus	22	−60	−10	2	0.017
**Cluster 2 (4 contributing subject groups, volume: 960 mm^3^, weighted center: *x* = −36, ***y*** = **7**, ***z*** = −14)**					
Left superior temporal gyrus	38	−36	2	−18	0.016
Left insula	13	−36	12	−10	0.014
**Cluster 3 (3 contributing subject groups, volume: 960 mm^3^, weighted center: ***x*** = 42, ***y*** = −1, ***z*** = 2)**					
Right claustrum		40	−2	2	0.019
Right insula	13	48	4	2	0.010
**Cluster 4 (3 contributing subject groups, volume: 952 mm^3^, weighted center: *x* =** −**56, *y* =** −**32, *z* =** −**13**)					
Left middle temporal gyrus	21	−58	−30	−16	0.015
Left middel temporal gyrus	20	−54	−36	−10	0.013
**Cluster 5 (3 contributing subject groups, volume: 856 mm^3^, weighted center: ***x*** = **15**, ***y*** = −66, ***z*** = 26)**					
Right precuneus	31	16	−66	26	0.016
**Cluster 6 (3 contributing subject groups, volume: 840 mm^3^, weighted center: ***x*** = 9, *y* = −51, ***z*** = 46)**					
Right precuneus	7	8	−52	46	0.016
**Cluster 7 (3 contributing subject groups, volume: 656 mm^3^, weighted center: ***x*** = **27**, ***y*** = **6**, ***z*** = −3)**					
Right putamen		30	4	0	0.013
Right putamen		24	6	−6	0.011
**Cluster 8 (2 contributing subject groups, volume: 616 mm^3^, weighted center: *x* = 15, *y* = −25, *z* = −2)**					
Right thalamus		14	−26	−2	0.016
**Cluster 9 (3 contributing subject groups, volume: 608 mm^3^, weighted center: *x* = −53, *y* = −24, ***z*** = **7**)**					
Left superior temporal gyrus	41	−54	−24	6	0.015
**Cluster 10 (4 contributing subject groups, volume: 584 mm^3^, weighted center: *x* = −4, *y* = −18, *z* = 4)**					
Left thalamus (medial dorsal nucleus)		−4	−16	6	0.012
Left thalamus		−6	−22	−2	0.010
Right thalamus		4	−20	6	0.009
**Cluster 11 (2 contributing subject groups, volume: 528 mm^3^, weighted center: ***x*** = 22, ***y*** = 62, ***z*** = 12)**					
Right superior frontal gyrus	10	22	62	10	0.012
Right superior frontal gyrus	10	22	62	8	0.011
Right superior frontal gyrus	10	30	60	6	0.009

*p < 0.05 with cluster-based thresholding to correct for multiple comparisons, coordinates reported in MNI space, anatomical labels representing nearest gray matter locations, contributing subjects groups only denotes groups with original foci located within the resulting cluster*.

### Increased spontaneous functional connectivity/activity in MDD

Findings in group B mainly comprised the pre-/subgenual anterior cingulate cortex and neighboring medial frontal cortex, the precuneus and neighboring posterior cingulate cortex, lateral prefrontal cortex bilaterally with a left predominance, left lateral parietal cortex as well as the right hippocampus and right cerebellum. Detailed results are presented in Figures 1B,C and Table 4. For thresholded ALE-maps see the Supplementary Material.

**Table 4:** **Brain areas (cluster-information and peak voxels) with significant convergence across studies in Group B (increased connectivity/activity in depression)**.

**Anatomical label**	**BA**	**(Sub-)Maxima coordinates**			**ALE**
		***x***	***y***	***z***	
**Cluster 1 (3 contributing subject groups, volume: 1792 mm^3^, weighted center: ***x*** = **1**, *y* = −63, ***z*** = **41**)**					
Left precuneus	7	2	−56	44	0.015
Left cuneus	7	−2	−70	38	0.014
**Cluster 2 (4 contributing subject groups, volume: 1704 mm^3^, weighted center: *x* = −43, ***y*** = **25**, *z* = **24**)**					
Left middle frontal gyrus	46	−48	26	18	0.012
Left middle frontal gyrus	9	−38	24	28	0.011
Left middle frontal gyrus	9	−40	26	22	0.010
**Cluster 3 (3 contributing subject groups, volume: 928 mm^3^, weighted center: *x* = −42, ***y*** = −39, ***z*** = **52**)**					
Left inferior parietal lobule	40	−42	−40	52	0.013
**Cluster 4 (2 contributing subject groups, volume: 896 mm^3^, weighted center: *x* = −3, ***y*** = **56**, ***z*** = −18)**					
Left medial frontal gyrus	10	−6	56	−16	0.012
Left medial frontal gyrus	10	0	60	−20	0.009
Right medial frontal gyrus	10	2	56	−18	0.008
**Cluster 5 (2 contributing subject groups, volume: 736 mm^3^, weighted center: *x* = 6, *y* = 33, *z* = −10)**					
Right anterior cingulate cortex	24	6	34	−10	0.015
**Cluster 6 (2 contributing subject groups, volume: 688 mm^3^, weighted center: *x* = −44, *y* = −42, *z* = −36)**					
Left cerebellum (Anterior Lobe, Culmen)		−44	−42	−36	0.015
**Cluster 7 (2 contributing subject groups, volume: 680 mm^3^, weighted center: ***x*** = **33**, ***y*** = −34, ***z*** = −4)**					
Right hippocampus		32	−34	−4	0.015
**Cluster 8 (2 contributing subject groups, volume: 680 mm^3^, weighted center: ***x*** = **15**, ***y*** = −58, ***z*** = **23**)**					
Right posterior cingulate cortex	31	16	−56	24	0.011
Right precuneus	31	14	−66	22	0.008
**Cluster 9 (2 contributing subject groups, volume: 664 mm^3^, weighted center: ***x*** = −43, ***y*** = **39**, ***z*** = **8**)**					
Left middle frontal gyrus	46	−44	38	8	0.011
**Cluster 10 (2 contributing subject groups, volume: 648 mm^3^, weighted center: ***x*** = **57**, ***y*** = **22**, ***z*** = **17**)**					
Right inferior frontal gyrus	9	56	22	18	0.015
**Cluster 11 (2 contributing subject groups, volume: 624 mm^3^, weighted center: ***x*** = **43**, ***y*** = −45, ***z*** = −42)**					
Right cerebellum (Tonsil)		44	−44	−42	0.012
**Cluster 12 (2 contributing subject groups, volume: 624 mm^3^, weighted center: ***x*** = −5, ***y*** = **34**, ***z*** = **12**)**					
Left anterior cingulate cortex	24	−6	34	12	0.013
**Cluster 13 (2 contributing subject groups, volume: 592 mm^3^, weighted center: ***x*** = **34**, ***y*** = **31**, ***z*** = **28**)**					
Right middle frontal gyrus	9	34	30	28	0.013

*p < 0.05 with cluster-based thresholding to correct for multiple comparisons, coordinates reported in MNI space, anatomical labels representing nearest gray matter locations, contributing subjects groups only denotes groups with original foci located within the resulting cluster*.

## Discussion

The main purpose of this meta-analysis was to provide spatially precise information about locations of altered FC or local brain activity in patients with MDD compared to healthy controls. We will therefore first discuss how the resulting data can be used in subsequent studies including applications with diagnostic intention. This particularly refers to FS for diagnostic MVPA approaches.

Additionally, subordinate aspects related to the results will be discussed: this study is not primarily designed to elucidate the exact functional nature of alterations of spontaneous brain activity. However, in order to estimate what aspects of the disease mechanisms may be captured based on our results, it is important to understand how they relate to other neurobiological and particularly neuroimaging findings. Therefore, results will be compared with other functional imaging approaches and with findings from structural neuroimaging.

### Potential applications

Results presented here can be treated as accessible prior knowledge about spatial locations of altered spontaneous brain activity in MDD. In particular this includes definition of regions of interest (ROIs) for hypothesis-driven group comparisons and particularly for FS (Pereira et al., 2009; Chu et al., 2012; Mwangi et al., 2013) in diagnostic classification efforts based on rs-fMRI data. For approaches using correlation based on seeds or pairs of regions of interest (Margulies et al., 2010), coordinates from Tables 3, 4 can be used directly. In addition, most software tools for voxel-based classification facilitate masking for FS (Schrouff et al., 2013). Therefore, NIfTI-files of thresholded ALE-maps in MNI space are provided (Supplementary Material).

In terms of classical RSNs several clusters of altered FC in MDD observed here correspond either to well-known midline structures as DMN subregions (Fox et al., 2005; Smith et al., 2009) or lateral frontal areas within a fronto-parietal network (Smith et al., 2009) associated with cognitive control (Niendam et al., 2012). It is therefore conceivable to use representations of established functional networks in the brain instead of the original results with the intention to enhance biological plausibility of analyses. Classical resting state networks (RSNs) cannot be quantitatively related to results of this meta-analysis with sufficient validity. Neither one of these single networks seems to comprehend “all” major meta-analytical findings nor do these point toward all major subregions of these networks in a qualitative comparison (see Supplementary Material for further details) despite the good correspondence of several single regions. Results may thus rather represent interactions between classical RSNs.

An exploratory qualitative comparison with spatial representations of recently introduced temporally independent functional modes (TFMs) of spontaneous brain activtiy (Smith et al., 2012) indicates a potentially better correspondence with meta-analytical results (Supplementary Material). However, we do not recommend selecting features for diagnostic classification in MDD based on single RSNs or TFMs at this point because there is only limited evidence for this available so far. This issue warrants further research.

To summarize, this analyses provides results intended to improve conceivable diagnostic classification approaches. But one should keep in mind that it cannot be concluded from the results that rs-fMRI will be clinically applicable in MDD.

### Comparison of results with additional neurobiological findings in MDD

#### Functional neuroimaging and functional implications

Several studies of rs-fMRI in MDD did not fulfill the inclusion criteria, mostly because of missing coordinate data, but relate to the main meta-analytic findings. Outstanding examples of these studies are discussed here: in a study using independent component analyses (ICA) (Li et al., 2013) a distinction of the DMN into an anterior and a posterior component was addressed. Both showed increased FC before treatment. Differences in the posterior DMN were normalized after antidepressant treatment, while abnormal FC persisted within the anterior DMN (Li et al., 2013). This distinction potentially relates to the fact that only one of these components was significantly identified across studies. Zhang et al. adopted graph theoretical measures to study the topological organization on networks in MDD. Patients exhibited increased nodal centralities, predominately in the caudate nucleus and DMN as well as reduced nodal centralities in occipital, orbitofrontal and temporal regions (Zhang et al., 2011). In another recent rs-fMRI study, published after the date of study identification for this analysis, Sambataro et al. also highlight a differential involvement of DMN subsystems in MDD: patients exhibited increased connectivity of ventral, posterior and core DMN components. The interplay from the anterior to the ventral DMN subsystems was reduced (Sambataro et al., 2013). These findings are in line with meta-analytically observed increases in spontaneous activity in some but not all DMN subregions.

Brain activity at rest has also been studied using positron emission tomography (PET) or single-photon emission computed tomography (SPECT) (Fitzgerald et al., 2008; Hamilton et al., 2012; Sacher et al., 2012). In contrast to rs-fMRI analyses these studies rarely adopt FC measures. In an ALE meta-analysis Fitzgerald et al. report a complex pattern of predominantly frontal alterations of brain activity featuring medial frontal hypoactivity, heterogenous findings regarding directionality of alterations in lateral frontal areas in both cerebral hemispheres and hyperactivity in the thalami (Fitzgerald et al., 2008). There is a fair spatial overlap with rs-fMRI findings but the directionality of alterations is not directly comparable. However, in an exclusive analysis of only four studies using 18F-Fluorodeoxyglucose-PET, regionally increased glucose metabolism was observed near the subgenual ACC (Sacher et al., 2012), an area of increased activity/connectivity observed by rs-fMRI. As a notable aspect, early results of single PET studies of increased brain activity in MDD in the subgenual ACC, orbitofrontal cortex, ventrolateral prefrontal cortex, thalamus as well as the amygdala and less-markedly even medial parietal areas who have substantially informed current integrated neurocircuitry models of mood disorders (Price and Drevets, 2010) exhibit a better correspondence with rs-fMRI results than the meta-analytic reports of PET-studies in MDD. Thus, it seems desirable to investigate in further studies if features selected by rs-fMRT itself are better suited than PET-derived features that have been used in previous MVPA studies in MDD, for example (Craddock et al., 2009). However, this issue cannot be finally resolved at the moment as there is no consensus regarding optimal classification algorithms for diagnostic purposes (Sundermann et al., 2014a).

Generally the functional organization of the brain at rest highly resembles networks involved in responding to specific tasks (Smith et al., 2009). Despite a host of task-based fMRI studies in MDD so far, results cannot be summarized in one coherent model. Meta-analyses and systematic reviews on altered emotion and cognition in MDD by task-based neuroimaging exhibit moderate (Fitzgerald et al., 2008; Stuhrmann et al., 2011) or poor (Delvecchio et al., 2012) spatial overlap with findings in rs-fMRI reported here and no consistent alterations of activity in posterior DMN components or the subgenual ACC were reported. Thus, rs-fMRI seems to be better suited to depict these systems presumably involved in MDD pathophysiology. On the other hand, alterations of amygdala activity were not consistently observed in rs-fMRI. It has been highlighted, that even the directionality of amygdala activity is highly dependent on the emotional valence of stimuli (Groenewold et al., 2013). Therefore, this dynamical aspect of potential disease mechanisms in MDD may not be sufficiently captured by potential diagnostic classification efforts based on spontaneous activity only. Thus, relying on amygdala activity diagnostically may complicate the differentiation of patients with anxious comorbidity, which is an important symptom in a subset of patients with MDD (Kupfer et al., 2012).

Based on functional imaging results the involvement of the brain areas mainly altered during wakeful rest in MDD pathogenesis has been discussed as follows: the abnormal interplay of cortical midline structures associated with self-referential processing, emotion-related brain areas and lateral cortical areas related to higher cognitive processing has been functionally interpreted as a correlate of pathologically increased ruminative brooding in MDD. In particular, a reduced top-down inhibition of cortical midline and limbic regions has been discussed (Marchetti et al., 2012; Nejad et al., 2013).

Further lines of function-related research in MDD focus on genetic and metabolic alterations including neurotransmitters and therapeutic interventions (Kupfer et al., 2012). Findings of this meta-analysis can however not be directly related to these efforts because of the heterogeneity of samples and methods in the original studies. Still, knowledge of general relationships between rs-fMRI and neurotransmitter-systems may help further elucidate pathogenetic mechanisms in MDD in the future (Barkhof et al., 2014).

#### Structural neuroimaging

There are repeated reports about specific regional volume reductions in MDD affecting the basal ganglia, hippocampus, frontal lobe (including the orbitofrontal cortex) and less consistently the cingulate cortex and thalamus (Koolschijn et al., 2009; Lorenzetti et al., 2009; Kempton et al., 2011; Arnone et al., 2012; Sacher et al., 2012). These reported locations, based on anatomical descriptors, resemble a subset of findings in rs-fMRI, a strict formal comparison is not feasible as results were mostly not reported in a common coordinate space. Posterior midline structures, central locations of aberrant spontaneous brain activity in MDD, do not seem to be significantly affected by these volume reductions.

White matter microstructure as an important aspect of suspected network pathology in affective disorders has been studied using diffusion tensor imaging (DTI) and derivative techniques: reduced anisotropy measures, a potential marker of fiber integrity, were observed in parts of the superior frontal white matter presumed to connect the dorsolateral prefrontal cortex and anterior cingulate cortex with subcortical nuclei (Sexton et al., 2009), the superior longitudinal fasciculus and increased anisotropy in the fronto-occipital fasciculus in MDD (Murphy and Frodl, 2011). The subgenual ACC associated with increased spontaneous activity/connectivity in this meta-analysis was identified as a potential site for therapeutic deep brain stimulation in MDD (Mayberg et al., 2005; Johansen-Berg et al., 2008; Lozano et al., 2008; Coenen et al., 2011). The structural connectivity of this area has been investigated using diffusion imaging as well, demonstrating widely distributed connectivity with frontal, limbic and visceromotor brain regions. An associated connectivity-based parcellation of the perigenual ACC revealed two distinct subdivisions, the pre- and the subgenual ACC (Johansen-Berg et al., 2008). The subgenual ACC observed in this meta-analysis of rs-fMRI data corresponds well with the latter location defined by distinct structural connectivity features (orbitofrontal cortex, medial temporal lobe and through the fornix) (Johansen-Berg et al., 2008).

Results of functional and structural imaging in MDD seem somewhat contradictory: some areas with increased spontaneous activity/functional connectivity seem to exhibit volume reduction or are served by white matter tracts with decreased anisotropy. Though functional and structural connectivity metrics show mostly concordant variations (Damoiseaux and Greicius, 2009; Honey et al., 2010), there are other examples of a similar paradox, e.g., in multiple sclerosis (Hawellek et al., 2011).

### Limitations of the current analysis

The analysis predominantly provides information about spatial congruency of resting-state fMRI findings in depression. It does, however, not allow estimation of effect sizes. Information about the directionality or further details of mechanisms of supposed alterations of functional connectivity is limited. This is particularly caused by the significant heterogeneity of different post-processing methodologies used in the studies reviewed. While interpretation of directionality in most of these methods is well-established for the so-called default mode network (Van Dijk et al., 2010) this does not necessarily generalize to other networks. The number of studies with highly analogous methods was not sufficiently high to facilitate method-specific meta-analyses with adequate statistical power.

The ALE-approach adopted here relies on sufficiently reliable studies reporting results in terms of whole brain coordinates. Thus, not every study reporting relevant group comparison results based on rs-fMRI data in MDD could be included for this methodological reason. In seed- or ROI-based analyses (Margulies et al., 2010) the original seed coordinates less strictly reflect the spatial location of potentially associated alterations and could therefore not be included in this coordinate-based analysis. This may limit the sensitivity for alterations in such regions that have been regarded of special importance by the authors of the original studies. The generalizability of results to other samples is also limited by the heterogeneity of samples in the studies included as these range from first-episode medication naïve subjects to treatment resistant patients after multiple depressive episodes. However, the literature currently available does not seem to facilitate a more specific meta-analysis regarding these features yet. As stated above this meta-analysis primarily pursued a methodological goal and therefore emphasized spatial specificity.

All aforementioned aspects potentially reduce the overall statistical power of this meta-analysis. The number of so-called “contributing” subject groups—actually the number of subjects groups with coordinates within a resulting cluster is however not a straightforward marker of statistical power in this setting as there is a complex relationship with cluster size. Figures 1A,B jointly depict statistically significant results and the distributions of raw ALE values in both groups. This gives an impression of the heterogeneity of the original results in both groups. This heterogeneity implies that alterations of FC or spontaneous brain activity are not a highly robust finding across different samples and methodological choices. This has to be taken into account when trying to make conclusions about actual disease mechanisms based on these results. In contrast to an earlier meta-analysis of rs-fMRI in MDD (Kühn and Gallinat, 2013), the main purpose of this study was feature selection for conceivable diagnostic classification by MVPA. To briefly reiterate, this means to identify a set of brain areas that contain potentially discriminative information to differentiate MDD patients from controls. Such feature selection is intended to reduce data dimensionality and discard irrelevant information in order to improve the diagnostic accuracy of classification approaches (Pereira et al., 2009; Mwangi et al., 2013; Sundermann et al., 2014a). Aggregating prior knowledge from the literature is a commonly applied feature selection step (Chu et al., 2012; Schrouff et al., 2013). Type II errors in such a preparatory meta-analysis can be much more problematic than limited type I errors. Missing important brain areas discriminating subjects with MDD from controls might reduce diagnostic accuracy. Owing to the (however not unlimited) ability of classification algorithms to identify and highlight most discriminative information (Pereira et al., 2009), a limited amount of false positive results in the definition of regions of interest or masks could potentially be better compensated for. There is no established standard for setting multiple comparison correction thresholds in ALE analyses yet as it is highly dependent on the number of studies and data distribution (Eickhoff et al., 2012). This issue is however much more problematic in meta-analyses that are primarily intended to elucidate disease mechanisms.

Multiple reports based on the same or similar data and overlapping samples are a generic problem in meta-analyses (Littell et al., 2008). In this work a recent modification of the ALE method (Turkeltaub et al., 2012) was adopted to minimize within-group effects of potentially overlapping samples without sacrificing valuable information. Despite that, it cannot be fully excluded that there is residual overlap of samples in studies considered independent here. However, we adopted a consensus based approach involving three reviewers to reduce this potential bias.

Even despite this issue the recent literature on rs-fMRI in MDD displays a noticeable tendency toward particular Asian as well as North American or European populations. As prevalence and clinical symptomatology differ significantly between cultural contexts (Kirmayer, 2001; Halbreich et al., 2007; Juhasz et al., 2012; Yeung and Chang, 2014) results reported in this meta-analysis may not necessarily be applicable to other populations.

Due to the different informational content a quantitative comparison with RSNs and TFMs, such as a formal conjunction analysis, was not feasible.

This meta-analysis focused on comparisons of depressive subjects and healthy controls. However, it seems to be even more desirable to identify differential neuroimaging biomarkers that provide information about individual prognosis or guide therapeutic decisions (Mossner et al., 2007; Sundermann et al., 2014a). Feature (pre-)selection for such efforts may be optimized specifically in the future as soon as further rs-fMRI research in these situations becomes available.

## Conclusion

This meta-analysis of resting-state fMRI studies in depression has identified a distributed pattern of brain regions with increased or decreased spontaneous activity compared to healthy controls. The most distinct finding is hyperactivity or hyperconnectivity presumably reflecting the interaction of midline structures (particulary posterior DMN components associated with self-referential processing and the subgenual ACC) with lateral frontal areas related to externally-directed cognition. Alterations that can be captured by rs-fMRI seem to differ from those identifiable with other neuroimaging modalities but show considerable overlap. Results of this meta-analysis are provided as coordinates and detailed maps in MNI space to be readily applicable for ROI selection in further rs-fMRI studies in MDD including feature selection for classification approaches with diagnostic intention. By emphasizing spatial precision and sensitivity this approach only provides limited information about the exact functional meaning of altered spontaneous brain activity in MDD.

## Author contributions

Benedikt Sundermann and Bettina Pfleiderer conceived and designed the study. Mona Olde lütke Beverborg and Benedikt Sundermann identified and screened the articles. Benedikt Sundermann, Bettina Pfleiderer and Mona Olde lütke Beverborg participated in final study selection and group assignment. Benedikt Sundermann and Mona Olde lütke Beverborg conducted the ALE-analyses. Benedikt Sundermann, Bettina Pfleiderer and Mona Olde lütke Beverborg participated in interpretation of the results. Benedikt Sundermann drafted the manuscript. All authors critically revised the manuscript for important intellectual content and approved the final version.

### Conflict of interest statement

The authors declare that the research was conducted in the absence of any commercial or financial relationships that could be construed as a potential conflict of interest.
