# Supplementary material for: Toward literature-based feature selection for diagnostic classification: a meta-analysis of resting-state fMRI in depression
Source: Front Hum Neurosci. 2014 Sep 10;8:692. doi: 10.3389/fnhum.2014.00692 (PMC4159995; doi:10.3389/fnhum.2014.00692)
Supplement: Supplementary file 2 [file Image1.PDF]

Supplementary Material

Towards literature-based feature selection for diagnostic classification: A meta-analysis of resting-state fMRI in depression

Benedikt Sundermann[1]*, Mona Olde lütke Beverborg[1], Bettina Pfleiderer[1]

[1]Department of Clinical Radiology, University Hospital Münster, Münster, Germany

*** Correspondence:** Dr. med. Benedikt Sundermann, University Hospital Münster, Department of Clinical Radiology, Albert-Schweitzer-Campus 1, Gebäude A1, 48149 Münster, Germany
benedikt.sundermann@ukmuenster.de

Supplementary Data

Output_GrA_ALE_C05_1.nii. Thresholded ALE maps of meta-analytical results in group A ($p < 0.05$, cluster-based correction) representing mainly decreased connectivity / function in depression.

Output_GrB_ALE_C05_1.nii. Thresholded ALE maps of meta-analytical results in group B ($p < 0.05$, cluster-based correction) representing increased connectivity / function in depression.

1. An exploratory qualitative comparison of meta-analytic results with resting state networks (RSNs) and temporally independent functional modes (TFMs) of spontaneous brain activity

Maps of meta-analytic results in MNI space were overlaid and visually compared with maps of published RSNs and TFMs (Smith et al., 2012) based on publicly available data from the WU-Minn Human Connectome Project (1U54MH091657), funded by the 16 NIH Institutes and Centers that Support the NIH Blueprint for Neuroscience Research. Correspondence of RSNs or TFMs with meta-analytic results was judged by consensus of BS and BP.

Single local maxima of the meta-analytic results corresponded with single RSN subregions, mainly posterior aspects of the DMN and lateral frontal areas with fronto-parietal RSNs. However, there was no RSN that spanned larger aspects of the meta-analytic results. In contrast one TFM, TFM 21 in the original publication (Smith et al., 2012), exhibited a good correspondence with a larger set of meta-analytically derived clusters from group B (Figure). This TFM comprises DMN areas anticorrelated with lateral-frontal regions often seen as part of an executive control or cognitive control network (Smith et al., 2012). Left lateral frontal areas and the posterior cingulate / precuneus cluster in group B representing increased connectedness in MDD correspond with positive connectivity in this TFM. In addition, the left parietal cluster in group B corresponds to a negatively correlated area included in the data on this TFM.

1.1. Supplementary Figure

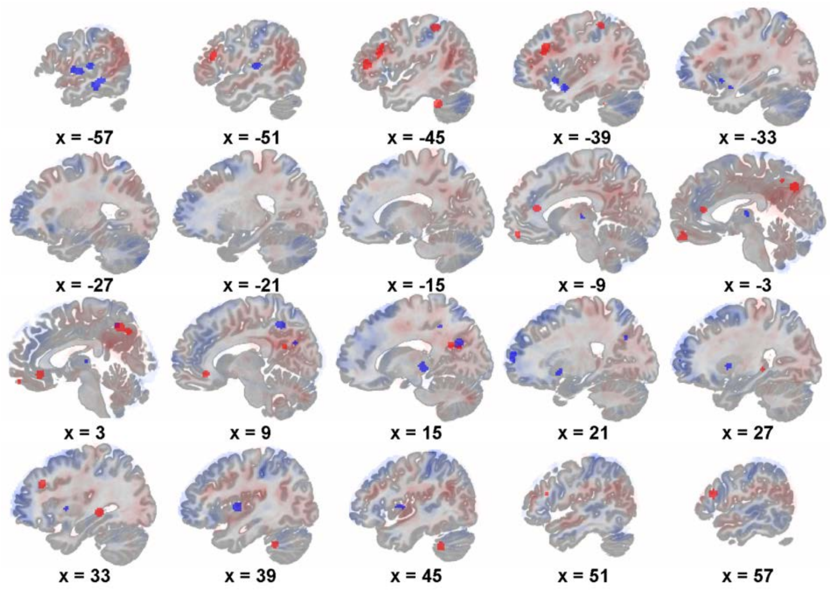

Supplementary Figure 1. Areas of altered functional connectivity / activity in depression ($p < 0.05$) compared to controls overlayed with TFM 21 (semi-transparent) from (Smith et al., 2012); blue: group A, red: group B.

2. References

Smith, S.M., Miller, K.L., Moeller, S., Xu, J., Auerbach, E.J., Woolrich, M.W., et al. (2012). Temporally-independent functional modes of spontaneous brain activity. Proc. Natl. Acad. Sci. U. S. A. 109, 3131-3136, doi: 10.1073/pnas.1121329109
